# Light-driven complex 3D shape morphing of glassy polymers by resolving spatio-temporal stress confliction

**DOI:** 10.1038/s41598-020-67660-9

**Published:** 2020-07-02

**Authors:** Jong Hyeok Lee, Jun-Chan Choi, Sukyoung Won, Jae-Won Lee, Jae Gyeong Lee, Hak-Rin Kim, Jeong Jae Wie

**Affiliations:** 1 Department of Polymer Science and Engineering, Inha University, 100 Inha-ro, Michuhol-gu, Incheon, 22212 Republic of Korea; 2 School of Electronics Engineering, Kyungpook National University, 80 Daehak-ro, Buk-gu, Daegu, 41566 Republic of Korea

**Keywords:** Polymers, Actuators

## Abstract

Programmable 3D shape morphing of hot-drawn polymeric sheets has been demonstrated using photothermal local shrinkage of patterned hinges. However, the hinge designs have been limited to simple linear hinges used to generate in-plane local folding or global curvature. Herein, we report an unprecedented design strategy to realize localized curvature engineering in 3D structures employing radial hinges and stress-releasing facets on 2D polymeric sheets. The shape and height of the 3D structures are readily controlled by varying the number of radial patterns. Moreover, they are numerically predictable by finite elemental modeling simulation with consideration of the spatio-temporal stress distribution, as well as of stress competition effects. Localized curvature engineering provides programming capabilities for various designs including soft-turtle-shell, sea-shell shapes, and saddle architectures with the desired chirality. The results of local curvilinear actuation with quantifiable stress implies options to advance the applicability of self-folded architectures embodying coexisting curved and linear geometric surfaces.

## Introduction

Self-folding of two-dimensional (2D) flat sheets is currently an area of interest for achieving programmable shape morphing into three-dimensional (3D) objects with minimized mechanical joints^1,2^. Recently, the transformation of 2D sheets into 3D geometry has been achieved using various external stimuli including light^3–7^, heat^1,8–11^, magnetic fields^12–16^, and chemicals^17–22^. In particular, bi-axially stretched thermoplastic polymers have been widely researched for self-folding due to the simple and low-cost actuation process used. This process is initiated by harmless near-infrared (NIR) light^23–27^. The bi-axially hot-drawn polymer sheets shrink at a temperature above that required for the polymer glass transition *T*_*g*_, by recovering random polymer conformation of pre-stretched polymer chains for entropy gain. Printing of ink patterns facilitates selective contraction of polymer films by the localized absorption of photonic energy in the printed regions. Through this photo-triggered strain engineering, high strain deformation of rigid glassy polymers has been demonstrated to generate angular structures^23,26^. Moreover, global curvature can be controlled by such as gradients of color^28^ and the density and coverage of inks^27^. Prediction of the photo-triggered 3D morphing has been achieved by analyzing the shrinkage effect of films only at hinge regions, and most previous research has been limited to control of simple folding-based shapes or to formation of gradually varying global curvature^23–25,28–31^.

In this study, we demonstrate a design strategy for facile construction of centro-symmetric or chiral 3D structures. This strategy is intended for engineering of local and global curvilinear strain by systematic investigation of stress convolution effects using simple radial hinge patterns and rigid facets. For example, programmable curvilinear morphing at a desired height is readily available by designing the size of facets and the number of hinges without intricate patterns, without gradients in material properties (i.e. crosslink density), and without patterned light irradiation. In order to achieve reliable 3D shape morphing, rigid facets were introduced as stress dissipation regions. The photo-triggered 3D morphing effects were systematically discussed according to pre-determined pattern conditions, such as the total number of hinges, symmetry of hinges, and the existence and size of the facets. Furthermore, we implemented finite element modeling (FEM) simulation with considerations of spatio-temporal stress distribution and their stress competition effects. The FEM simulation revealed hidden mechanisms of a strain re-distribution effect in non-patterned regions, which is associated with the structuring of the final 3D curvature. Interestingly, analytical computation using the FEM simulation results could be used to predict photo-triggered birefringence. This was confirmed by experimental visualization of the photo-actuated polystyrene (PS) films with polarizing optical transmittance images. Based on the systematic experimental results and the computational analysis for pre-designed hinge patterns, complex bio-mimetic 3D shapes could be successfully implemented. These included soft-turtle-shell and sea-shell shapes, for which the film actuation behaviours in the non-inked regions were as important as those in the inked, photo-actuating regions. Such actuation behaviours were also important in the formation of the final global 3D shapes. Furthermore, construction of saddle-like anticlastic architectures was achieved by selection of the desired chirality by introducing stress-releasing chiral patterns. The light-triggered manipulation of local curvilinear strain with quantifiable stress provides a significant step toward design of complex, self-foldable, 3D architectures with coexisting curved, and linear geometric surface. The selective shape morphing, thereby, offers potential opportunities in shape-reconfigurable^32^ and deployable^33^ structures, tunable photonic devices^34^, 4D printing^35^, and soft robotics^34^.

## Results

### Design of radial hinge patterns on bi-axially pre-strained PS film

**Figure 1:**
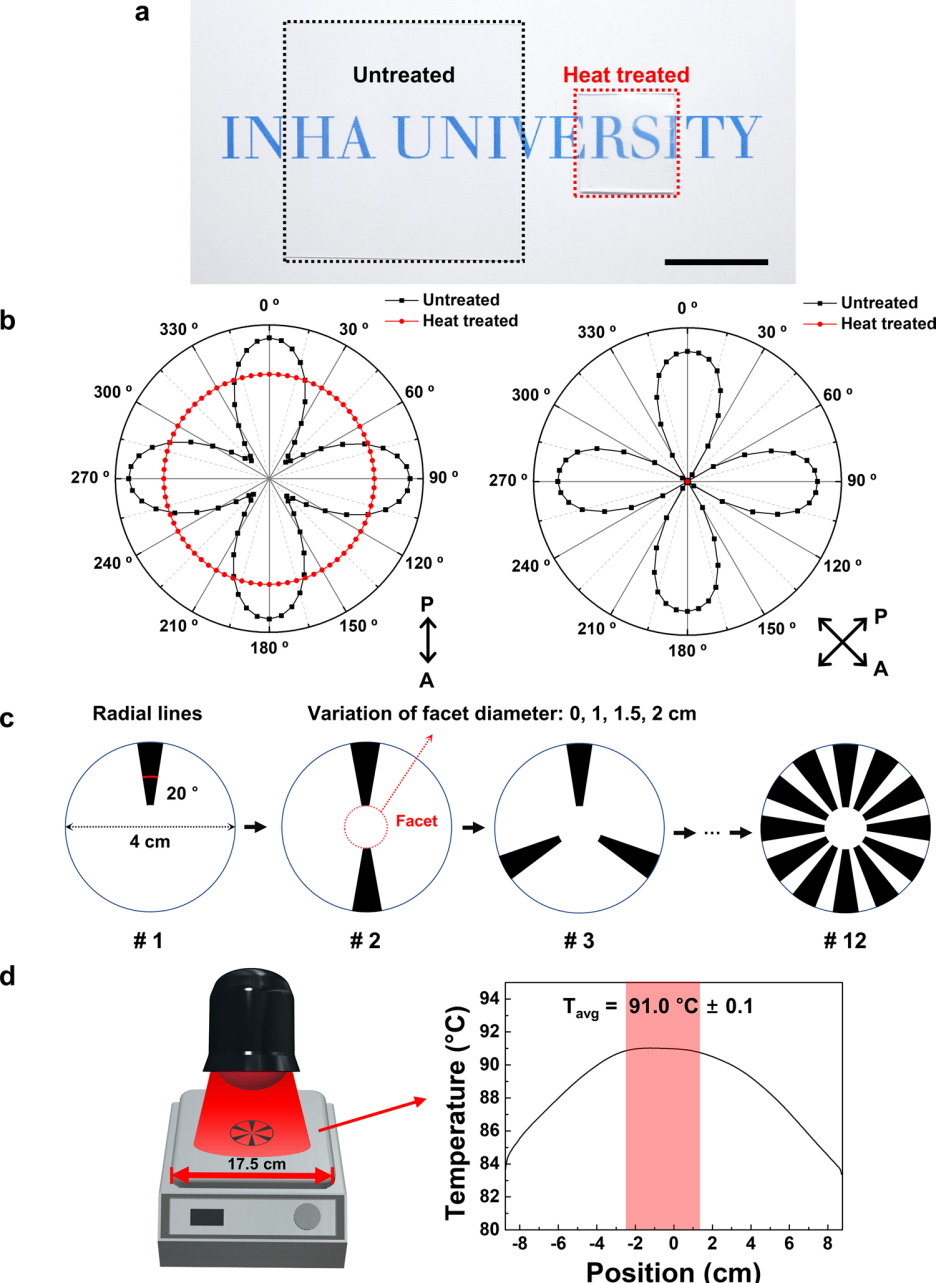
Design of the radial patterns and facets on bi-axially pre-strained PS films. (**a**) Global shrinkage of the pre-strained PS sheet by heat treatment in an oven at 130 °C (scale bar: 2 cm). (**b**) Polarization map of bi-axially pre-strained PS film with parallel and crossed polarizers by optical microscope. (**c**) Schematic illustration of the radial patterns (the number of radial patterns from 1 to 12) and circular facet design (the diameter of circular facets from 0 to 2 cm). (**d**) Exposure of NIR light to 2D patterns for 20 s on the pre-heated hotplate at 90 °C. The graph indicates the temperature range of the center of the pre-heated substrate.

The variation of radial hinge patterns on the 2D bi-axially pre-strained PS sheets provided programmable 3D curvature morphing via the NIR (*λ* = 600 to 1,600 nm) induced photothermal effects at the hinges. When the glassy pre-strained PS was heated above the PS *T*_*g*_ (~ 105 °C), PS sheets were subjected to shrinkage stress to recover a random polymer conformation due to entropy penalty. Undesired thermally conducted deformation of the non-inked region was minimized due to a large temperature deviation between the hotplate setting temperature (90 ºC) and high *T*_*g*_ of PS (105 ºC) as well as a fast heating rate by NIR exposure. Decent deformation of radial inked patterns in the bi-axially pre-strained polymer sheet was demonstrated in the hotplate-assisted photothermal system. With heat treatment in a 130 °C convection oven, an entire 5 × 5 cm^2^ polymer sheet exhibited 60% linear shrinkage (84% areal shrinkage), as shown in Fig. 1a. The drastic segmental change in conformation is visualized in Fig. 1b by presenting the optical transmittance of the PS films (before (■) and after (●) thermal treatment), with rotation of the films between parallel and crossed polarizers. Ideally, a PS film stretched along a bi-axially orthogonal direction with uniform strain should be optically isotropic for normal incident light without optical transmittance under the crossed polarizer condition. This should be true for any angle of rotation of the film. However, Fig. 1b shows that the commercially available pristine PS film has retardation that caused it to exhibit optical anisotropy. This might originate from the discrepancy between the amount of bi-axial stretching that occurred during manufacture of the PS film. The optical axes of the birefringent bi-axially pre-strained pristine film were parallel with the pre-strained directions (*S*_*px*_ and *S*_*py*_). After the thermally induced process of PS-film shrinkage was completed, the birefringent characteristics disappeared, as evident from the isotropic optical transmittance for both parallel and crossed polarizers. This dramatic optical change is indicative of the release of residual stress within the PS film during the dimensional shrinking at the polymer *T*_*g*_^23–25,27–31,36^. In our work, the birefringent behaviour of the pre-strained PS film was utilized as a visual tool for analyzing the morphing of the 3D curvature during photo-triggered strain engineering, which will be discussed in detail.

In our experiment, bi-axially pre-strained PS films were cut into circles with diameter of 4 cm, of which the uniform NIR intensity was secured from the Gaussian profile of the beam spot. On the flat PS films, inked radial patterns were printed with a general desk-top printer to demonstrate the localized heat-induced shrinkage effect. To investigate systematically the effects of localized shrinkage on centro-symmetric circular 3D curvature morphing, the number of the radial hinges was varied from 1 to 12, as shown in Fig. 1c. The angle of each hinge was 20°, regardless of the number of hinges. Hence, the surface coverage density of hinge patterns along the radial direction remained constant at a fixed radial hinge number while the inter-hinge distance was minimum at the center of the PS film. Moreover, the overall surface coverage density of the hinges over the entire surface area, systematically increased with higher pattern numbers. To explore the stress convolution and relaxation from the hinges and facets, we also introduced a non-inked round facet pattern at centers of varied diameter (*D* = 0, 1, 1.5, or 2 cm).

For contactless photothermal actuation, unpolarized NIR at 0.4 W cm^−2^ was used to irradiate the ink-patterned PS film while it was on a hotplate at elevated temperature. Before the NIR light exposure, the samples were pre-heated on the hotplate for 30 s for thermal equilibrium. In our experiment, the hotplate was set at 100 °C, but the actual surface temperature measured at the top layer of the pristine PS film by a forward-looking infrared (FLIR) camera was 91 °C (Fig. 1d), indicating ambient cooling. Under this condition, in the case of a PS sheet without an inked pattern, the thermally induced shrinkage effect did not occur after NIR irradiation, as shown in Supplementary Fig. 1. This is because the film temperature did not reach the PS *T*_*g*_ (~ 105 °C). Conversely, the temperature of the PS sheet was elevated to over 110 °C when the black ink was printed over an entire PS sheet due to effective NIR absorption by the black ink. The spatially resolved surface temperature profile was recorded after 2 s of NIR irradiation. Despite the aforementioned uniform NIR irradiation conditions, the surface temperature distribution has a clear gradient because of heat conductance and heat capacitance effects within the film, as well as the effect of heat dissipation into the ambient air. Without introducing any specifically designed hinge patterns, after 9 s of NIR irradiation, the PS film exhibited crumpling behaviour that originated from non-uniform thermal and stress convolution effects.

### Centro-symmetric curvilinear 3D curvature morphing with photo-triggered strain engineering

**Figure 2:**
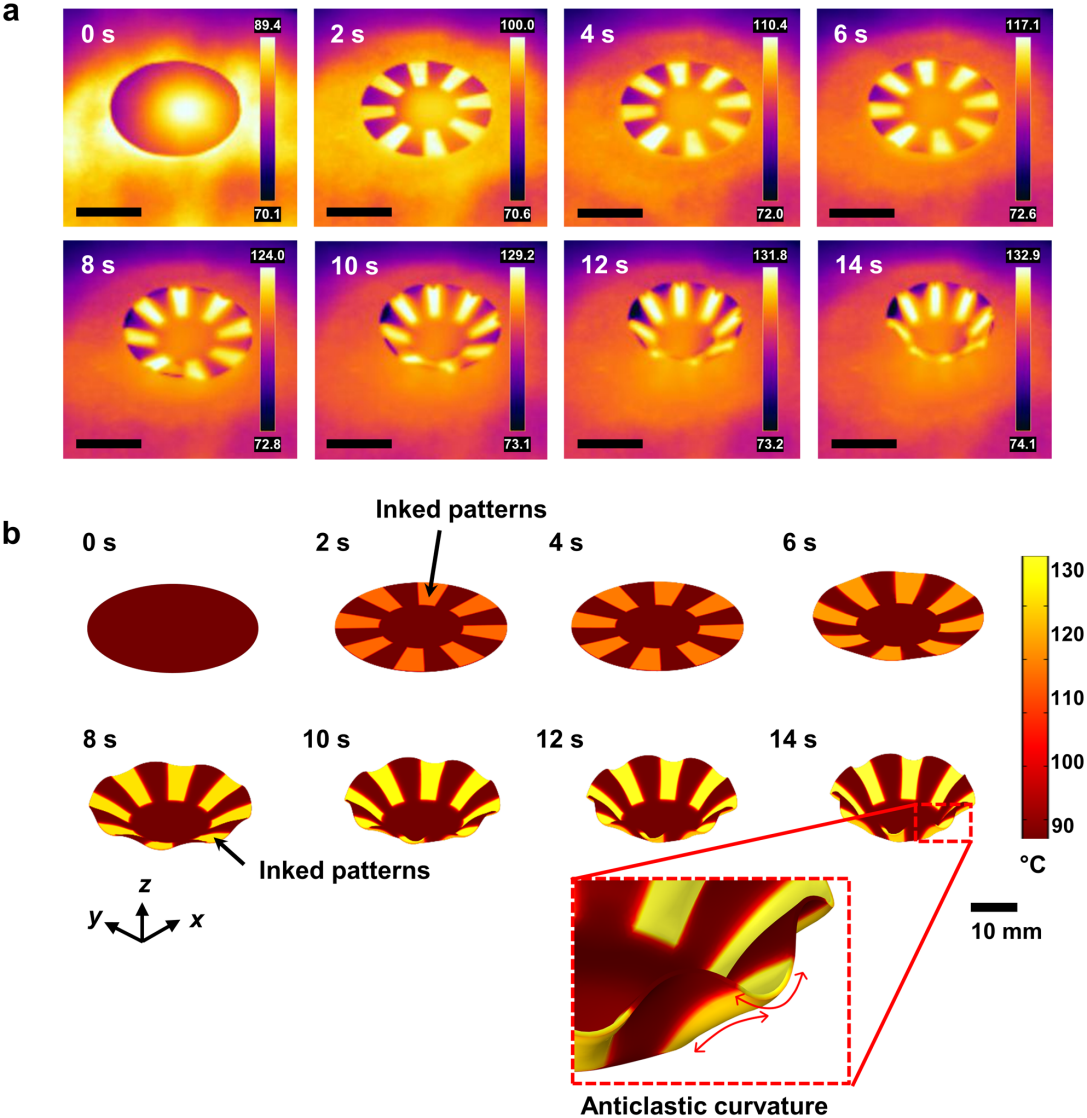
Time-resolved 3D shape morphing by photo-triggered curvilinear strain engineering. (**a**) Experimental results of the thermal images obtained by the FLIR camera (the scale bar: 2 cm). (**b**) FEM simulation results showing the temporal shape morphing and surface temperature distribution. Shown is PS film with a radial pattern (*N* = 8) and a non-patterned central facet region (*D* = 2 cm). The enlarged final image is presented to describe the formation of anticlastic curvature in the patterned regions by spatial stress competition effects between patterned and non-patterned regions. See Supplementary Movie 1 in the Supporting Information.

As shown in Fig. 2a, the time-resolved thermal images of the PS sample show controlled 3D shape morphing with 8 radial hinge patterns and a 2 cm central round facet. The PS film was placed on the hotplate and the patterned surfaces were subjected to an effective local increase in temperature due to the NIR irradiation, as evident from the FLIR camera results. However, NIR-induced shape morphing was not observed during the initial 4 s of NIR irradiation. After continuous NIR irradiation over 4 s, the surface temperature of the ink-patterned regions exceeded the PS *T*_*g*_ while non-inked regions still had surface temperatures below the PS *T*_*g*_. This localized glassy-rubbery phase transition resulted in localized shrinkage effects beneath the inked patterns, which drove the 3D shape morphing. The thermal shrinkage stress of the hinges was restricted along the radial direction by the adjacent glassy non-inked regions. Due to this interface effect, the thermal shrinkage of the hinges was more effective along the tangential direction than along the radial direction. Accompanying this interface-induced anisotropic shrinkage effect within the alternating patterned regions, the non-inked central facet area made a significant contribution to the generation of reliable 3D curvilinear deformation by preserving rigid stress-dissipating regions during folding-up of the radially patterned regions. The final structure from 3D-morphing was obtained after NIR irradiation for 14 s. The surface temperature of non-inked regions was measured to be up to 93 °C, lower than the PS *T*_*g*_. This was a centro-symmetric circular 3D shape, unlike the result for the PS film sample with ink printed over its entire surface (see Supplementary Fig. 1).

To understand the mechanisms of dynamic 3D shape morphing, analysis of the spatially resolved stress effects is essential in order to account for the tension applied at the non-inked regions. This is induced by the anisotropic shrinkage effect occurring in the adjacent patterns. This spatial stress competition effect at interfaces results in temporal non-linear strain evolution for 3D curvilinear shape morphing. In the previous case of simple, linear folding-based, shape morphing^23–25,28–31^, the effects from such spatial stress competition could be ignored because the dimensions of the linear hinge regions were highly localized, causing negligible conflict in the spatial stress.

To model the dynamic variation of complex 3D curvilinear morphing, we established an FEM method that considered the effects of spatio-temporal stress tensor reorganization within the shape morphing film. At every simulation step (using fine time intervals of 0.25 s), the relative spatial stress tensors were computationally obtained with respect to the previous simulation time step. During this computation, a finite amount of the temperature-dependent localized shrinkage effect was considered to be the perturbation source determining the temporal 3D shape morphing of the next time-step in the simulation. At each step, temperature re-distribution within the film was analyzed along the lateral and depth directions by accounting for heat conductance, heat capacitance, and heat dissipation effects toward the environmental boundaries. Based on the spatial temperature distribution computation, the temperature-dependent material parameters were updated for each time step of the simulations. More detailed information on the simulation conditions is presented in the Materials and Methods section. Similar to the experimental results, the 3D shape morphing in the FEM simulation caused by NIR-induced local photothermal effects, started at the 4 s time mark (Fig. 2b). Despite the complex stress re-distribution effects within the PS film, the temporal shape evolution at 14 s predicted by the FEM model was in good agreement with the experimental results. For the sample with a non-inked central facet, the final structure by the simulation also exhibited a centro-symmetric circular 3D curvature. Furthermore, both simulation and experiments demonstrated opposite signs of curvature along the tangential direction of the radial axes at the hinge pattern regions, as shown in the zoomed-in image of Fig. 2b. Interestingly, an anticlastic curvature was formed below the inked regions. This was because of the stress competition effects at the aforementioned glassy-rubbery interfaces, along with increase in the Poisson’s ratio (approaching 0.5)^37^ by the localized heating above the *T*_*g*_. The dynamic 3D morphing shape (oblique view) and photo-triggered stress distribution (top view) produced by the simulation, are presented in Supplementary Movie 1.

### Mechanisms of even–odd effects of hinge patterns and rigid facets

**Figure 3:**
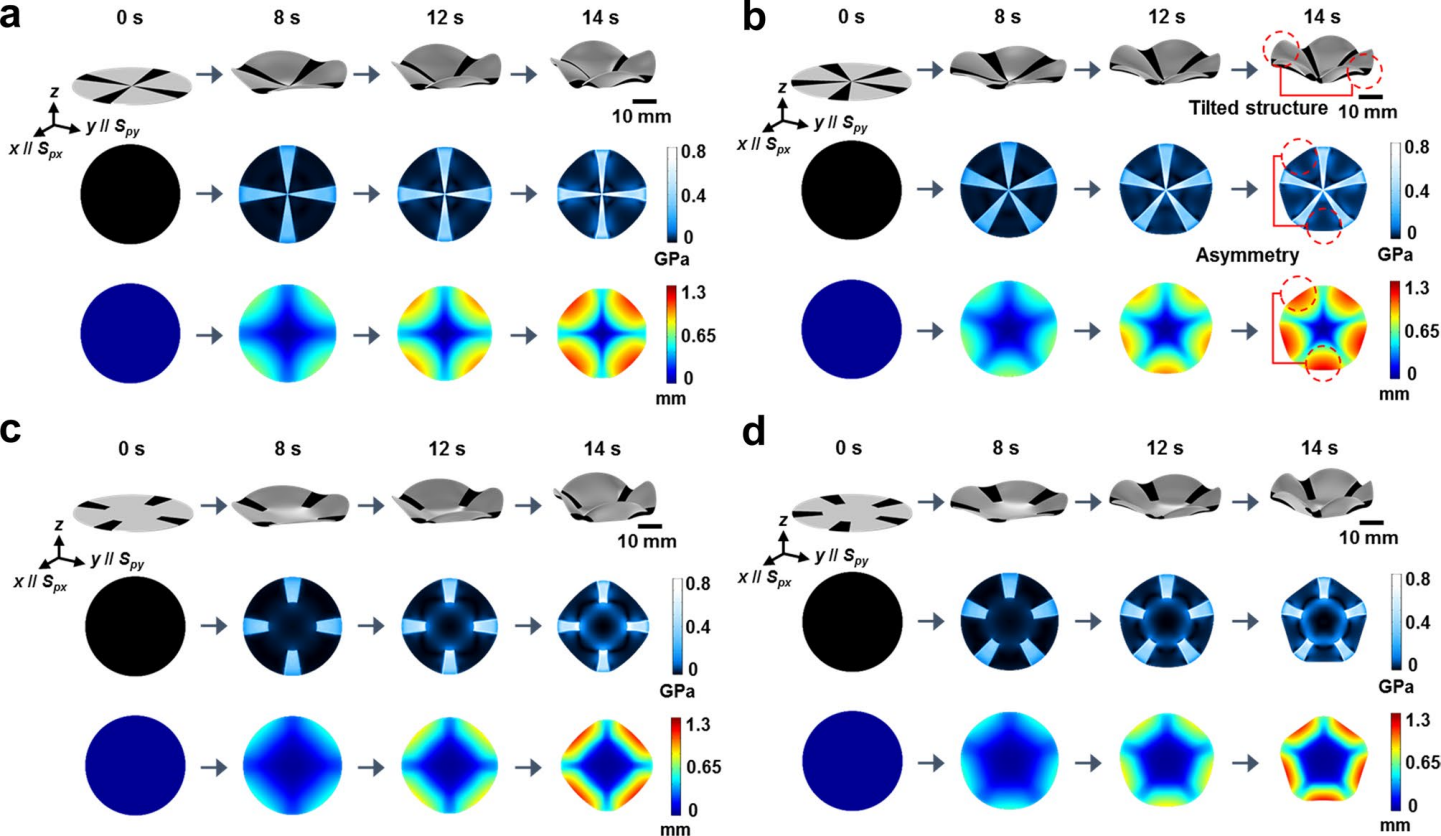
FEM simulation results of 3D curvature formation. (**a**)–(**b**) Four and five radial patterns without a central facet region. (**c**)–(**d**) Four and five radial patterns with central facet regions. Shown are even-numbered and odd-numbered radial patterns for samples with and without non-patterned central facet regions (*D* = 2 cm). For each set, there is an oblique view of the NIR-induced 3D shape morphing, top views of spatial stress maps, and a color map depicting the surface height distribution (top to bottom images, respectively).

Unlike the conventional narrow linear hinge folding mechanism, centro-symmetric circular 3D curvature morphing requires significantly larger hinge areas, resulting in complex temporal stress distributions associated with the final 3D curvature structure. To elucidate the effects of the pattern geometry on the morphing of the centro-symmetric circular 3D curvature, FEM simulations were performed according to the total number (*N*) of radial hinge patterns, which modify the relative coverage density of the hinge regions and inversion symmetry of the patterns (even–odd effects). The presence of the central facet was also considered (identical NIR irradiation conditions). Figure 3 shows the FEM simulation results for the pattern-dependent 3D curvature formation, depicting the time-resolved shape morphing upon exposure to NIR actinic light, corresponding stress map, and a color map for the surface height profile. Note that the pre-strained axes (*S*_*px*_ and *S*_*py*_) of the PS film, before NIR-induced actuation, were parallel to the *x*- and *y*-axis, respectively. Hence, the pre-strained axes were parallel with the radial pattern directions when *N* = 4. Without the central facet, the temporal and final 3D shape morphing that was actuated with the radial inked pattern of *N* = 4, was highly symmetric. This also resulted in symmetric stress and height distributions, as shown in Fig. 3a. However, the FEM results for *N* = 5 (Fig. 3b) show asymmetric behaviour in terms of strain, internal stress, and height distribution, as indicated with the dashed red circle in Fig. 3b. These observations can be further generalized to the even–odd effects of radial pattern numbers. As evident from the experimental results shown in Supplementary Fig. 2, the 3D shape morphing becomes more symmetric with an even-numbered pattern than with odd-numbered patterns owing to the nature of their inversion symmetry. However, the symmetry is no longer sustainable even for even-numbered patterns when the total pattern number increases. Unlike the computational results, the experimental conditions were not ideal, deviating from the uniform NIR irradiation, NIR-absorption, and temperature-dependent viscoelasticity typical of polymeric films. The FEM results shown in Fig. 3a and b indicate that the highly increased internal stress distribution was concentrated near the center of the round-cut PS film and generated asymmetric stress conflict in the odd-numbered pattern cases. In addition, the initial experimental pre-strained amounts between the bi-axial directions were different, as indicated in Fig. 1b, whereas the simulations assumed uniform PS film pre-stretch along the bi-axial directions. Thus, the temporal asymmetric deformations (in experiments) accumulated until the final structuring, which resulted in the catastrophic failure of 3D shape morphing in cases of larger hinge numbers. These non-ideal deviations became more severe for odd numbers of hinges than for even numbers of hinges.

**Figure 4:**
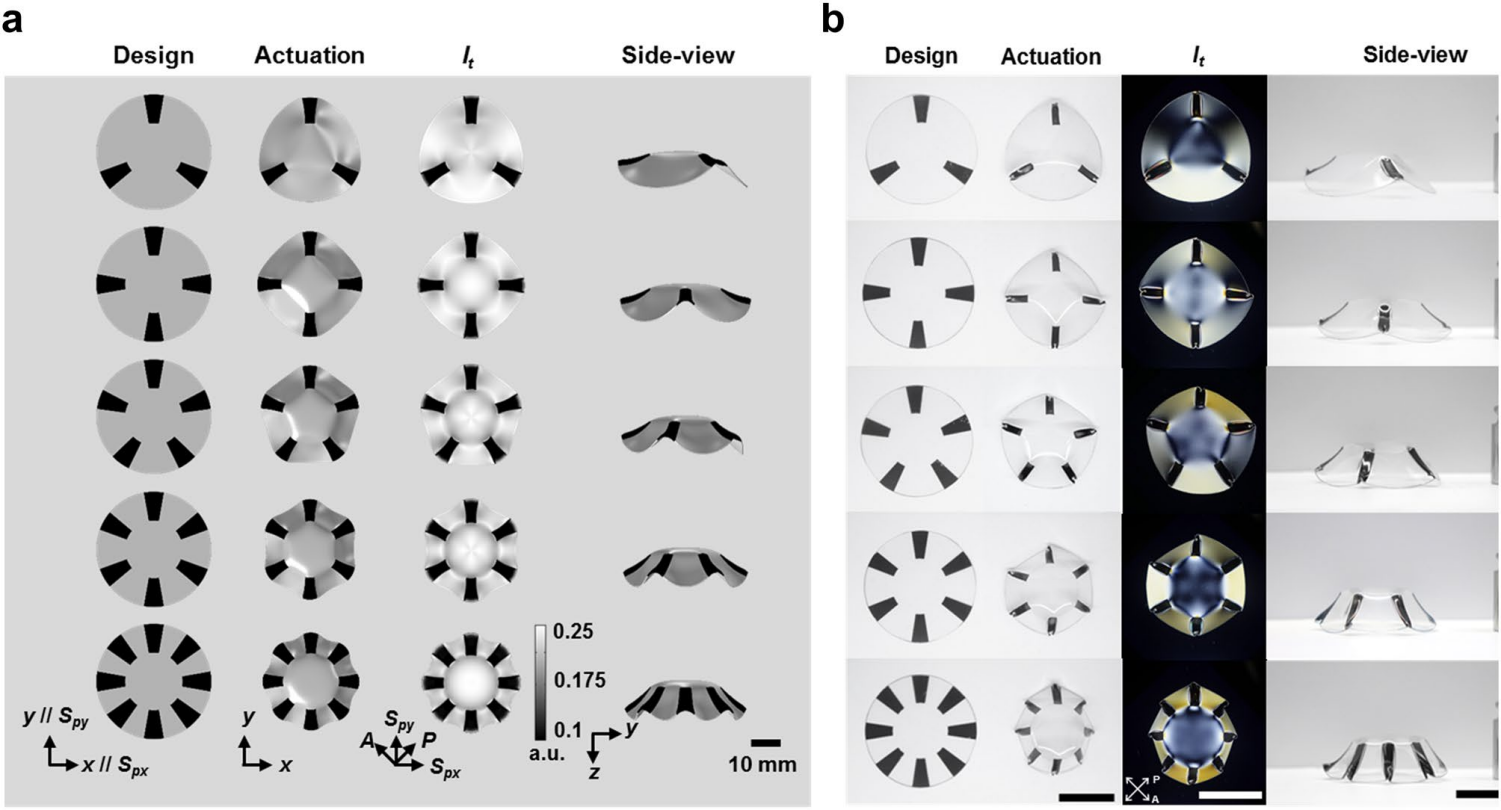
Three-dimensional curvature formation according to the number of radial patterns with facet. (**a**) FEM simulation results for each sample condition and (**b**) experimental results (from the left to right, the top view images of the ink-patterned PS films with non-inked central facet region before NIR-induced actuation; top view images of the NIR-actuated films. The optical transmittance (*I*_*t*_) images are as observed between crossed polarizers, and their side view images (of actuated samples turned upside down). From top to bottom, images are shown of samples with radial patterns with 3–8 arms with central facet region (*D* = 2 cm). The scale bar indicates 2 cm.

These undesired catastrophic failures could be mitigated by the introduction of central non-inked facets. As evident in Fig. 3c and d, the central stress is effectively dissipated around the glassy central facet region. Hence, the anisotropic shrinkage effects no longer generated highly concentrated stress convolution in the center region, resulting in morphing of the centro-symmetric circular 3D curvature. Importantly, symmetric behaviours could be obtained for both odd and even numbers of hinges. Similar to the computational analysis (Fig. 4a and Supplementary Fig. 3), the experimental results (Fig. 4b and Supplementary Fig. 2) also show more reliable symmetric curvilinear morphing for both types of hinges. Due to the practical experimental conditions, distorted final 3D shapes were obtained with the smaller facets, or with larger number of hinges slightly deviating from the simulation results. However, the centro-focused stress conflict could effectively be alleviated using a facet of greater diameter. The centro-symmetric 3D curvilinear morphing was thus viable for large hinge numbers, as evident from Supplementary Fig. 2 and Supplementary Movie 2.

As discussed for the computational and experimental results, the stress competition effects between the thermally actuating radial hinges and the non-inked regions, are extensively involved in the global shape control of the centro-symmetric 3D curvilinear morphing. The thermal actuated hinges generate tensile effects on the non-inked regions, and this causes re-distribution of the spatial strain axes in the non-inked regions as well. This was experimentally visualized using optical transmittance under crossed polarizers, as shown in Fig. 4b and Supplementary Fig. 4. In the experimental images, the incident beams at the hinges were blocked by the printed ink patterns, and the transmittance levels in the facet region and the non-inked radial pattern regions varied according to re-distributed strain axes from pre-strained axes. Under our experimental conditions, the optical transmittance in the non-inked regions decreased when the spatial stain axis was re-distributed towards the polarizer axis, which revealed a tensile stress effect by adjacent hinges.

Figure 4a and Supplementary Fig. 5 present the optical transmittance images produced by the simulation. To compute the spatial transmittance level, the re-distributed strain axes for both the hinges and the non-inked regions, were extracted from the FEM simulation results. In addition, the spatial retardation amounts were calculated, reduced from the initial PS film condition. As shown in Fig. 1b, after sufficient thermal shrinkage, the optical retardation of the PS films disappeared. For simplicity of simulation, we assumed a linear relationship between the spatial shrinkage in the FEM simulation and the reduced optical retardation (∆Γ<0). Then, the spatial optical transmittance (*I*_*t*_) could be expressed as follows^38^,

1 $$I_{t} \propto \sin^{2} (2\Phi ) \cdot \sin^{2} \left( {\frac{{\Gamma_{i} + \Delta \Gamma }}{2}} \right)$$

where *Φ* is the azimuthal angle between the polarizer axis and the re-distributed strain axis, and Γ_i_ (~ 1.59 rad obtained from experimental measurement) is the optical retardation of the initial PS film. In Supplementary Fig. 5, the ∆Γ-dependent gray map images indicate high decrease of retardation at the localized hinge regions by the NIR irradiation, although we assumed that there were heat-flow effects within the PS film. Except for the boundary regions of the hinges, the gray map image shows negligible ∆Γ variation in the non-inked regions. However, the gray map images depicting the *Φ*-dependent function value (Eq. 1), show that there are significant pattern-dependent variations in the non-inked regions, indicating close stress interaction between the non-inked and hinge regions. Remarkably, the final sets of computational optical transmittance images (Supplementary Fig. 5) matched well with the experimental results illustrated in Supplementary Fig. 4.

**Figure 5:**
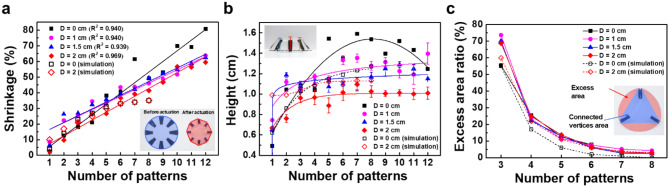
Analysis of radial hinge pattern effectiveness. (**a**) Shrinkage of shape-morphed films evaluated from a top-down view. (**b**) Height of shape-morphed films measured from side-view. (**c**) Excess area ratio of shape-morphed films calculated from the top-down view (*D*: diameter of the central facet).

We analyzed the covering shrinkage, height, and excess area ratio of 3D structures to evaluate quantitatively the effectiveness of the radial hinge patterns. Symmetric curvature depends on the number of radial lines and the inner facet diameter. When the initial area (*A*_*0*_) of the sample was reduced to *A* by NIR actuation, the shrinkage in percent (100 × (*A*_*0*_—*A*)/*A*_*0*_) was calculated using NIH ImageJ software, using a top-down image. Note that the initial sample area was 12.6 cm^2^ for all 4 cm circular PS samples. As evident from Fig. 5a, we confirm that the larger central facets provide enhanced linearity for the shrinkage against hinge numbers. The linearly proportional correlation is indicative of uniform shrinkage from each pattern by effective stress dissipation through the facets. Because the shrinkage from the top-down image is the source of the height increase during the 2D to 3D shape morphing, substantial shrinkage may correspond with the extreme height of an actuated sample as the number of hinges increases. The average height of the 3D structure was calculated from three points in a side-view image, as shown in Fig. 5b. As expected, the absence of a central facet resulted in both non-linear shrinkage-hinge number correlations, as well as to difficulty with height control. Moreover, asymmetric height was observed for the samples without facets, while the presence of larger central facets allowed more uniform height after actuation. Interestingly, the presence of central facets resulted in saturation of the height, which means that the height of the final 3D structure could be programmed using the facet diameter. Because the height increase resulted from out-of-plane actuation, this height saturation effect indicates greater in-plane shrinkage at higher numbers of hinges. The in-plane shrinkage was localized at the hinges and this shrinkage stress was mainly dissipated at the hinge-facet interfaces. Hence, the vertices of hinges produced polygon-shaped deformation of the circular facet. By connecting each vertex of the hinges, we obtained an area of polygons (*A*_*P*_) and the excess area (*A*_*E*_) needed to calculate the excess area ratio (percentage, represented as 100 × *A*_*E*_ /*A*_*P*_). As summarized in Fig. 5c, the excess area rapidly decreases with higher number of hinges. Therefore, by considering the discussions on shrinkage, actuated height, and excess ratio, uniform and symmetric polygonal shaping of initially circular facets was achieved, along with 3D structures of programmable height.

### Complex 3D shape morphing: bio-mimetic shapes and saddle shapes

**Figure 6:**
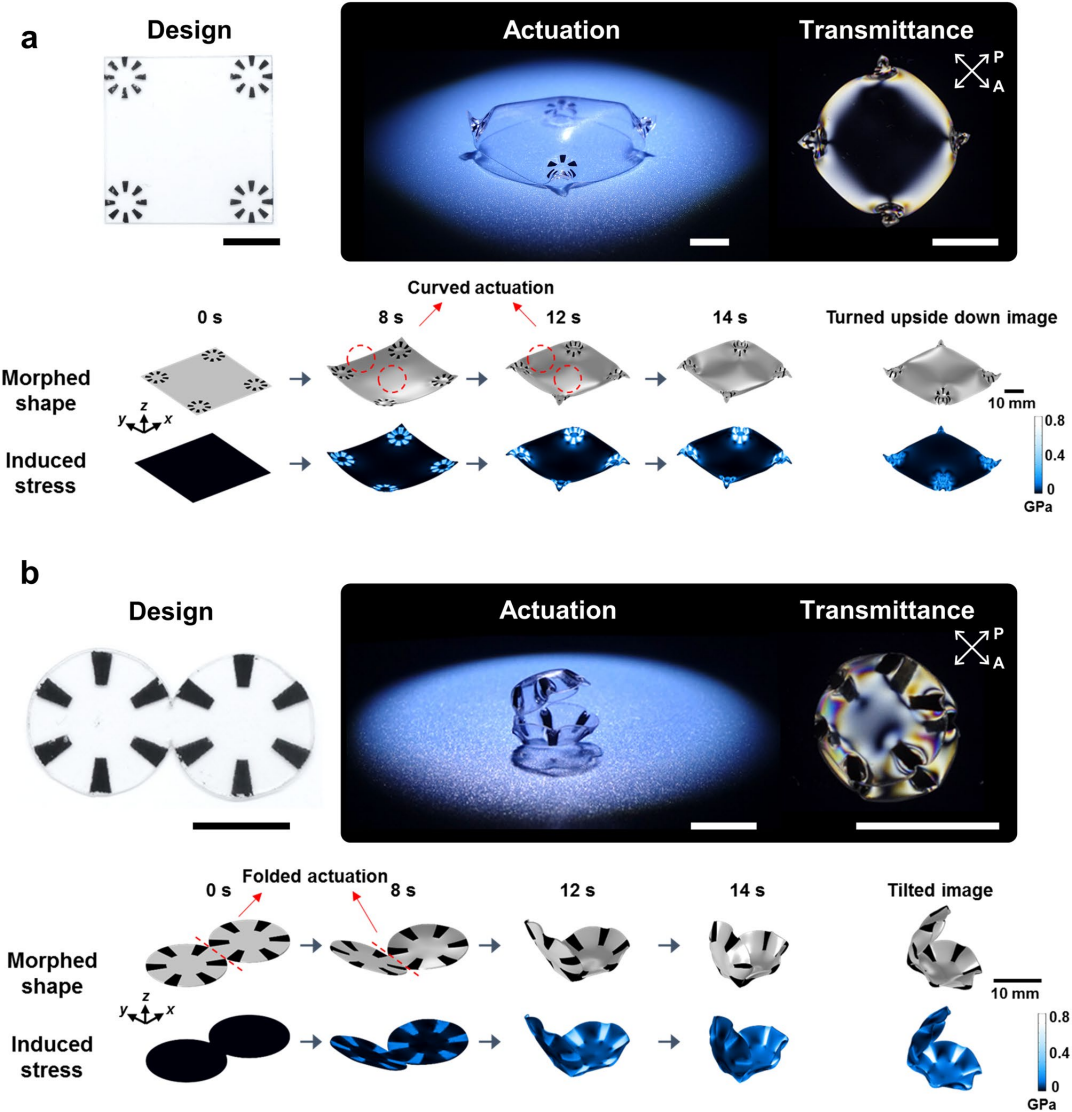
Construction of complex 3D shapes using localized curvilinear deformation with radial hinge patterns and facets. (**a**) Soft-turtle-shell structure and (**b**) sea-shell structure. See Supplementary Movies 3 and 4 of the time-resolved shape morphing and spatial-stress distribution of both samples. For each set, the upper images show the experimental samples before and after the NIR-induced actuation. The optical images with crossed polarizers are presented after actuation to visualize the re-distribution of strain axes. The bottom images are the FEM simulation results demonstrating temporal 3D shape morphing and induced stress distributions.

Based on the results from evaluating pattern effectiveness, we realized bio-mimetic 3D curvature morphing through combinations of patterns involving radial hinges and round facets. The pattern design shown in Fig. 6a has eight radial hinges printed at four corners of a square PS sheet (4 × 4 cm^2^). After uniform NIR irradiation, this sample was deformed via 3D self-structuring to form a soft-turtle-shell curvature. The top shell surface of the 3D-morphing final structure possessed a gradually varying curvature and its polarizing image also showed a highly symmetric optical transmittance pattern. In Fig. 6a, the FEM simulation results show time-resolved 3D morphing and the photo-triggered stress distribution. The example of the photo-triggered complex 3D morphing of a soft-turtle-shell structure shows that highly deformed shape morphing localized at four corners could also make 3D global-shaped architecture in the non-inked region.

**Figure 7:**
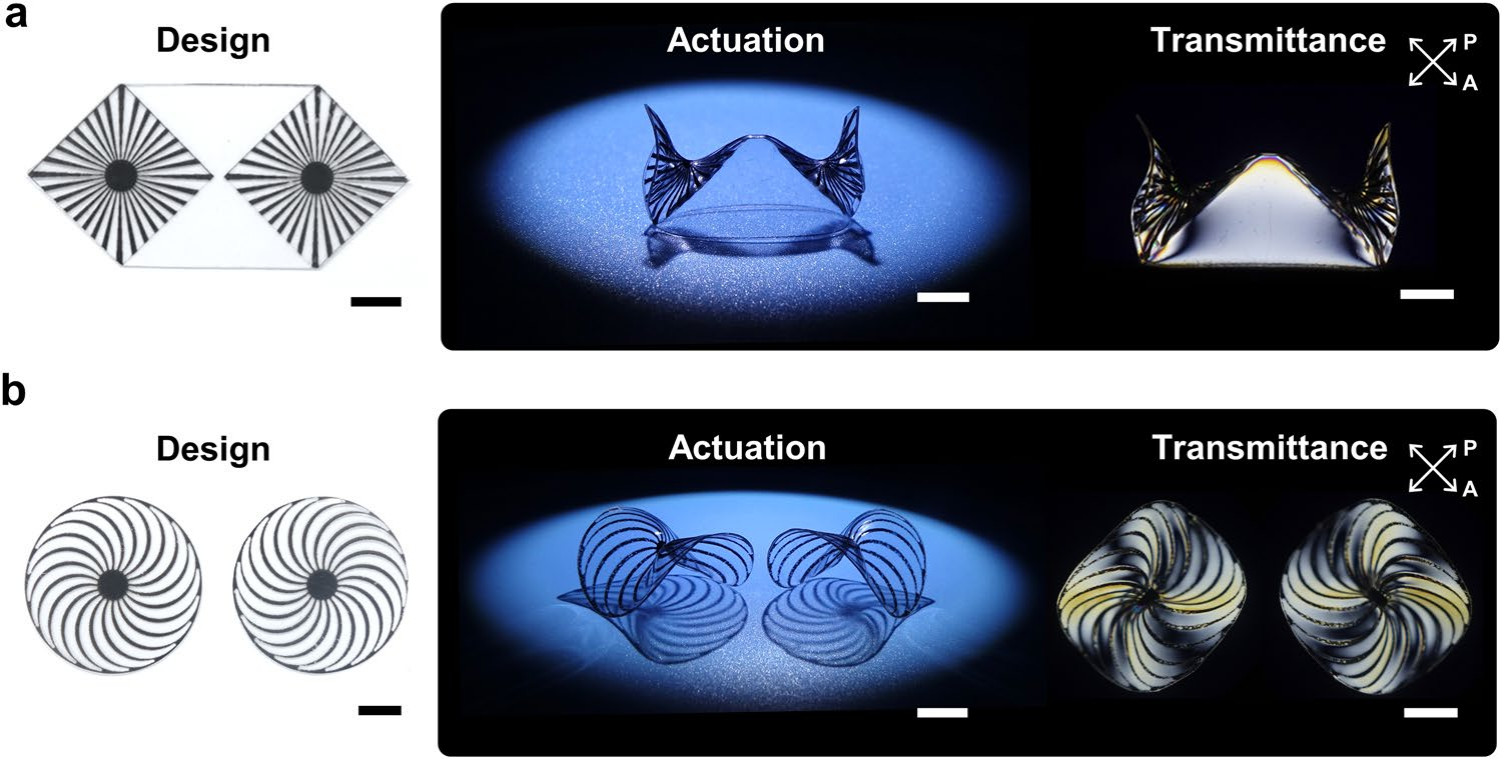
Construction of 3D saddle structures using localized curvilinear deformation with modified radial hinge patterns. (**a**) Achiral radial patterns to induce an achiral saddle (Viking helmet architecture) and (**b**) chiral radial patterns to induce a chiral saddle architecture (left: counter-clockwise, right: clockwise). The scale bars indicate 1 cm.

The radial hinge patterns (*N* = 6) and central facets (*D* = 0.75 cm) in Fig. 6b, were designed on two connected (1.5 cm diameter) circular boundary conditions for sea-shell 3D morphing. Interestingly, the global curvature formed in each round region and the folding-like actuation co-existed in the final sea-shell 3D morphing, as shown in the time-resolved simulations of Fig. 6b. Unlike the folding actuation achieved by the previous sharp line hinges^23–25,28–31^, the folding actuation in Fig. 6b occurred in the non-inked region, clearly demonstrating the effects of stress propagation from the hinge interfaces. Hence, consideration of the photo-triggered interfacial stress effect in the non-inked regions, is indispensable for explaining 3D morphing, as with the previously discussed radial patterns. The FEM analysis presented again provides excellent agreement with the experimental sea-shell 3D morphing result. For both types of bio-mimetic 3D complex-shape morphing, the time-resolved simulations are presented in Supplementary Movie 3 and 4. Finally, we introduced modified radial hinges for realization of more complicated 3D curvature engineering. Figure 7a depicts a design with modified radial patterns (32 lines, internal angle of line is 5°, interval of lines is 11.25°, and diameter of inner black circle is 0.33 cm) and a side-view image after actuation. Also shown is its optical transmittance image between crossed polarizers. In contrast with the single radial patterns discussed with Supplementary Fig. 2, Fig. 7a shows the interface regions between two radially patterned areas and two triangular facet areas. Thus, the photothermal shrinkage was restricted along the interfaces by the non-inked facets and the stress conflict at two centers of two radial patterns was released by forming a Viking-helmet-like 3D curvature during NIR-induced actuation (see Supplementary Movie 5). The edges of the radial patterns have larger deformation at the air interface, while larger radius of curvature was observed with facet interfaces due to the aforementioned stress dissipation. The triangular 3D morphing of the triangular facets suggests wide geometric potential for the design strategies using hinge and facet combinations.

In our previous pattern examples, we focused on self-structuring of the centro-symmetric 3D morphing by employing the stress-releasing facets. Figure 7b depicts a pre-designed scheme of the chiral radial hinge patterns introduced for resolving the stress-conflict effect. The hinge patterns have counter-clockwise or clockwise chirality consisting of 20 equiangular spirals with a 0.7 cm central black circle. After the photo-triggered engineering, the oblique images of Fig. 7b showed chirality selection of saddle-like 3D morphing, according to the chirality of the radial patterns. We discussed the effect of the localized anticlastic curvature formation in Fig. 2b. In Fig. 7b, Poisson’s effect resulted in the construction of global anticlastic curvature in the 3D shape having two orthogonal curvatures with opposite signs. Distortion of the chiral strain axis by the spiral hinges in the non-inked regions was observed, as evidenced by an optical transmittance image with crossed polarizers. The chiral saddle-like 3D morphing suggests that the introduction of chiral hinges provides an effective way of resolving the stress conflict effects during the photo-triggered 3D shape morphing from the 2D sheets. Consequently, we are assured that introducing a simple radial hinge pattern design is capable of realizing a variety of 3D curvilinear shape morphing.

## Discussion

We demonstrated photo-triggered curvilinear strain engineering to achieve complex 3D structures from 2D glassy polymeric films. As a model system, we employed radial hinges and circular facets by printing black ink on bi-axially pre-strained PS films for photothermal stress induction and distribution, respectively. The number of radial patterns and diameter of the stress-distributing facet allowed the control of the spatial stress, which affected the pattern shape via shrinkage, height, and excess area ratio. When the coverage density of the radial patterns was too large for localized photo-shrinkage, or if the odd numbered patterns were employed without facets, catastrophic failures were observed due to high stress interactions or asymmetric spatial stress, respectively. Due to the introduction of round facets, stress convolution effects were mitigated and the target centro-symmetric 3D shapes were successfully constructed, with selection of polygonal geometry and height. The final 3D structures predicted through the spatio-temporal FEM simulation were a good match with the experimental results that evolved dynamically during photo-actuation. To visualize the strain axis re-distribution effect that resulted from the spatial stress competition effects, we measured optical transmittance with crossed-polarizers, and again, successful prediction was achieved by the FEM simulation. By sophisticated design of hinge and facets using various boundary conditions, we implemented complex bio-mimetic 3D morphing that included soft-turtle-shell and sea-shell structures, in both experiments and FEM simulations. Furthermore, local and global anticlastic curvature engineering was achieved with the selection of achiral, clockwise, and counter-clockwise chirality for 3D saddle architectures. Photothermal morphing of the PS sheets secured resultant configuration owing to high *T*_g_. Consequently, we expect that deployable structures^33^ through curvilinear strain-engineering will be produced with consistency with use of commercial PS, based on the localized curvature predicted by FEM simulations. In addition, recoverable soft materials such as liquid crystal elastomers^39^, shape memory polymers^34^, and vitrimers^40^ will be eligible for reversible actuation. This curvilinear strain-engineering design strategy and spatio-temporal stress analysis will enable expanded complexity of actuation in applications requiring shape-reconfigurable scaffolds, tunable photonic devices, 4D printing, and soft robots.

## Methods

### Photo-triggered curvilinear strain engineering

Two-dimensional radial patterns were printed onto 0.25 mm-thick bi-axially pre-stretched PS sheets (Grafix, Shrink film KSF-C) using a desktop laser printer (HP MFP M277dw). The patterned PS sheets were cut into 4 cm-diameter circular shapes with a non-inked inner facet (facet diameter = 0, 1, 1.5, or 2 cm). The number of radial hinge patterns was systematically varied from 1 to 12. The NIR-triggered shape morphing of the PS sheets was conducted on a hot place at 100 °C (Misung Scientific, HS 180) after 30 s of equilibrium for temperature stabilization. The localized shrinkage of the PS sheets in the ink-patterned areas occurred upon exposure to 0.4 W cm^−2^ NIR light (UNIX, UIM-250) within 1 min, resulting in autonomous shape morphing into 3D architectures. The localized temperature elevation of the PS sheets was measured using FLIR camera (FLIR Systems, Inc.). The optimal NIR exposure of 14 s resulted in the 3D structure from the 2D polymer sheet without unexpected crumpling and/or burns from overheating (see Supplementary Fig. 6). Young’s modulus of PS glassy-rubbery region was measured in by dynamic mechanical analyzer (DMA) (see Supplementary Fig. 7).

### Spatio-temporal FEM simulation

The computational analysis of pattern-dependent 3D shape morphing was performed using an FEM simulation (COMSOL Multiphysics, COMSOL Inc.). The temporal evolution of the spatial stress tensor distribution and its dynamic 3D curvilinear deformation was calculated with time intervals of 0.25 s in order to consider the stress competition effect between the inked and non-inked regions, as well as the temperature-dependent material parameter variations during pattern-dependent selective heating. At each time step of the FEM simulations, the spatial temperature conditions for the hinges and non-inked regions were analyzed along the lateral and depth directions, considering the thermal conductivity and thermal capacitance of the PS film. In addition, the spatial thermal shrinkage effects and the stress-competition between adjacent mesh elements were obtained at each time step for calculation of the next time step of the simulation. Among the temperature-dependent material parameters, Young’s modulus and Poisson’s ratio were adopted from the material library of COMSOL Multiphysics. The temperature profiles of Fig. 2 show that non-inked regions still remain at glassy state up to the moment of the final 3D structuring but the inked regions undergo phase transition to a rubbery state. For the simulation, the Poisson’s ratio value varying from 0.347 at 25 °C to 0.410 at 135 °C is applied. The heat dissipation effects from the PS films toward the environmental boundaries were reflected by considering the air convection as a heat transfer coefficient of 100 W m^−2^ K^−1^ where the initial air temperature condition at each simulation time step of the FEM analysis was reset at 90 °C. Between each time interval of the FEM simulation, temperature re-distribution of the air near the PS film was computed. In consideration of these temperature-dependent simulation conditions, we are able to confirm that FEM results matched well with the experimental results of the photo-triggered curvilinear 3D morphing. The surface temperature profile of the inked layer was experimentally measured (see Supplementary Fig. 8) as a function of the time of exposure to NIR irradiation, and then applied in the FEM simulation.

## Supplementary information

Supplementary file1

Supplementary file2

Supplementary file3

Supplementary file4

Supplementary file5

Supplementary file6
