# Supplementary material for: Light-driven complex 3D shape morphing of glassy polymers by resolving spatio-temporal stress confliction
Source: Sci Rep. 2020 Jul 2;10:10840. doi: 10.1038/s41598-020-67660-9 (PMC7331612; doi:10.1038/s41598-020-67660-9)
Supplement: Supplementary file 1 — Supplementary file1 [file 41598_2020_67660_MOESM1_ESM.pdf]

Electronic supplementary information (ESI)

Light-Driven Complex 3D Shape Morphing of Glassy Polymers by Resolving Spatio-Temporal Stress Confliction

Jong Hyeok Lee,[‡a] Jun-Chan Choi,[‡b] Sukyoung Won,[a] Jae-Won Lee,[b] Jae Gyeong Lee,[a] Hak-Rin Kim, [*b] Jeong Jae Wie[*a]

[a.] *Department of Polymer Science and Engineering, Inha University, 100 Inha-ro, Michuhol-gu, Incheon 22212, Republic of Korea. E-mail: wie@inha.ac.kr*

[b.] *School of Electronics Engineering, Kyungpook National University, 80 Daehak-ro, Buk-gu, Daegu 41566, Republic of Korea E-mail: rineey@knu.ac.kr*

‡ *These authors contributed equally to this work*

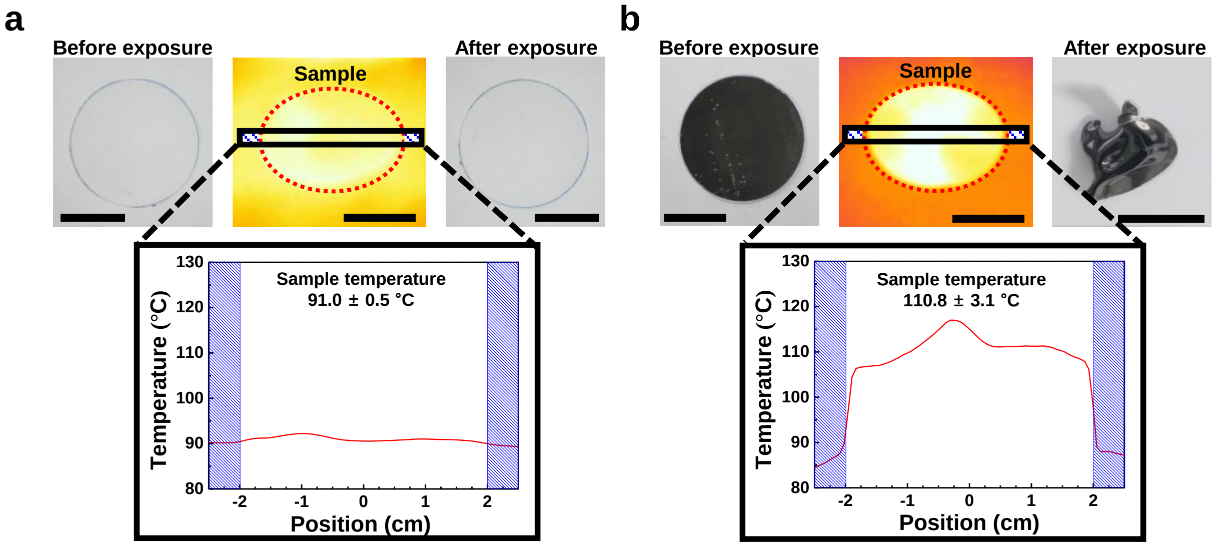

Supplementary Fig. 1 Photothermal effect of the PS film with or without printed ink over the entire surface. (a) Thermal imaging of PS film without total printed-ink-coverage, and its temperature variation (average temperature 91.0 ± 0.5 °C). (b) Thermal imaging of fully covered ink-printed PS film and its temperature variation (110.8 ± 3.1 °C); the temperature distribution was taken after 2 s of NIR irradiation and the collapsed structure is shown after 9 s of NIR irradiation. The scale bars indicate 2 cm.

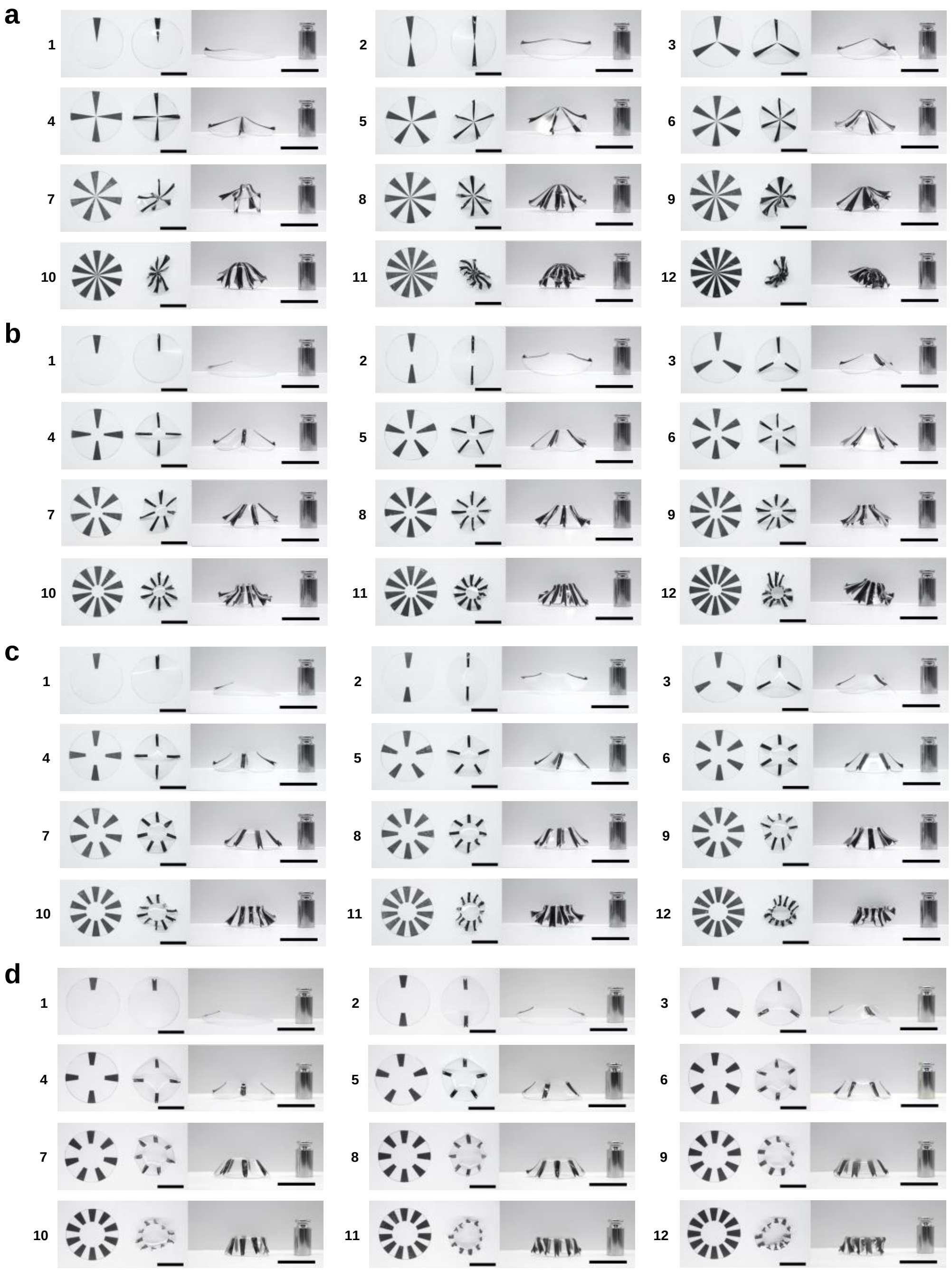

Supplementary Fig. 2 Experimental results of the NIR-actuated films according to the pattern types. (a) Radial patterns without a central facet and (b)–(d) radial patterns with central facets (*D* = 1, 1.5, or 2 cm, respectively). The top view images of the pristine film, the top view images of the NIR-actuated films, and the side view images of the NIR-actuated films are shown from left to right images for each experimental set. Note that the samples were manually flipped after actuation. The scale bar indicates 2 cm.

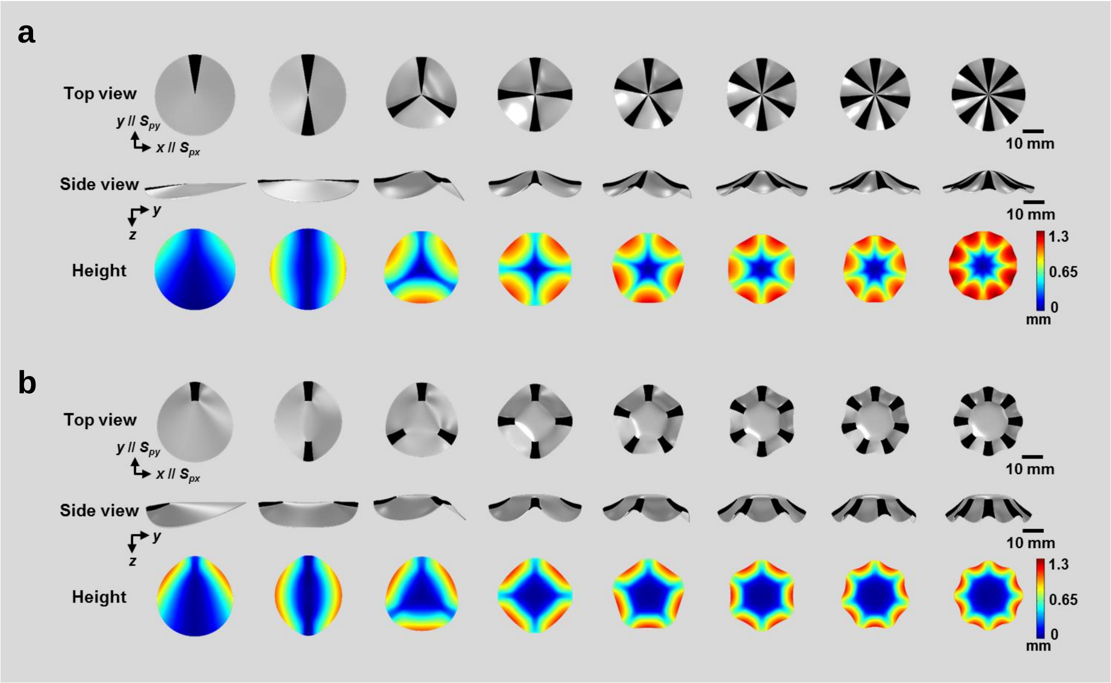

Supplementary Fig. 3 FEM simulation results of pattern-dependent 3D curvilinear shape-morphing. (a) Radial patterns without a central facet and (b) radial patterns with a central facet (*D* = 2 cm). The top and side view images are shown for the final 3D shape morphed by localized heating effect and resultant stress distribution. The color map images are of the surface height profiles.

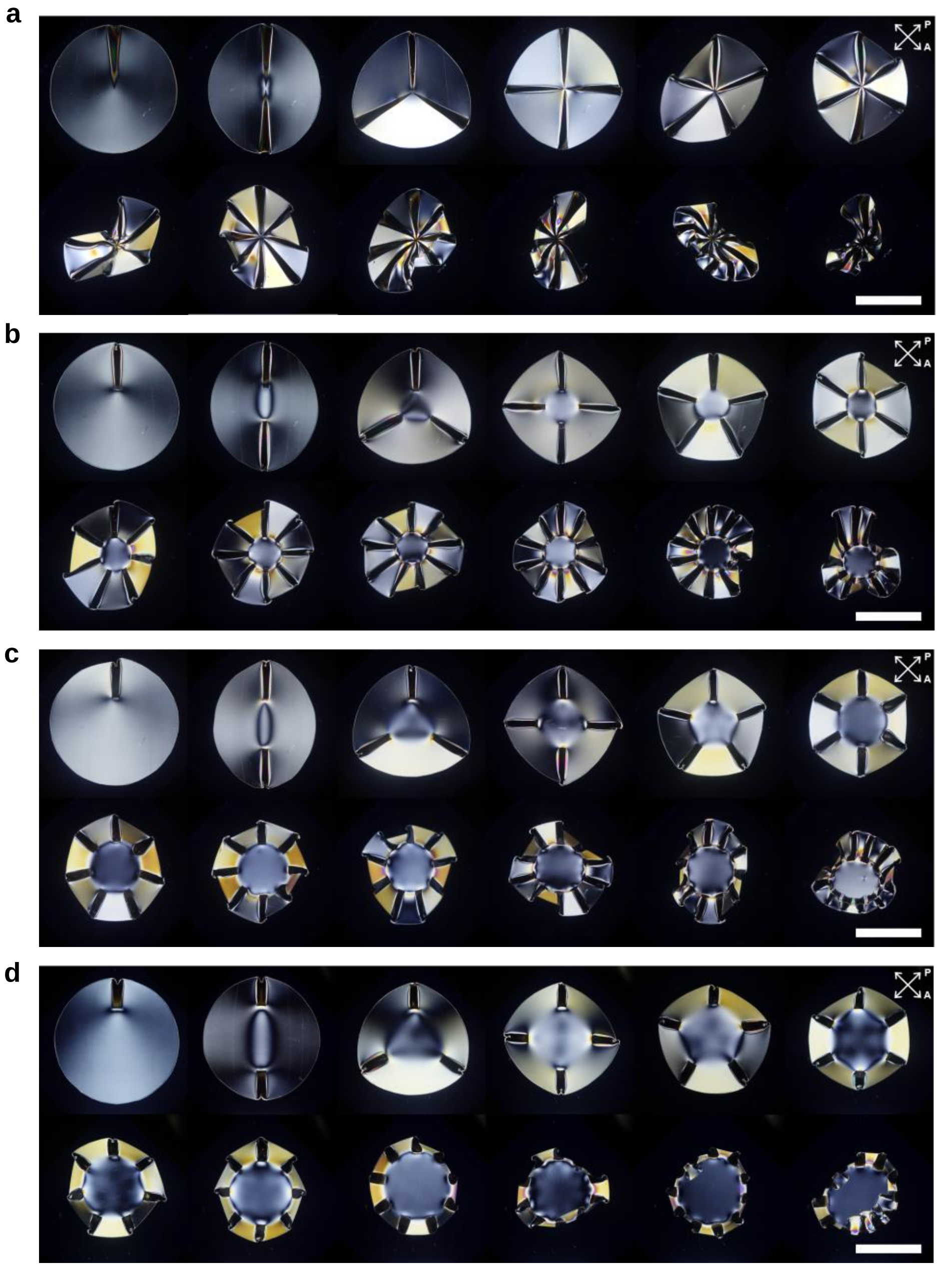

Supplementary Fig. 4 Experimental optical images of the NIR-actuated films on crossed polarizers. (a) Radial patterns without a central facet and (b)–(d) radial patterns with central facets (D = 1, 1.5, or 2 cm, respectively). This demonstrates the spatial light transmittance (I_t) to visualize re-distribution of the spatial strain axis according to the pattern types. The scale bar indicates 2 cm.

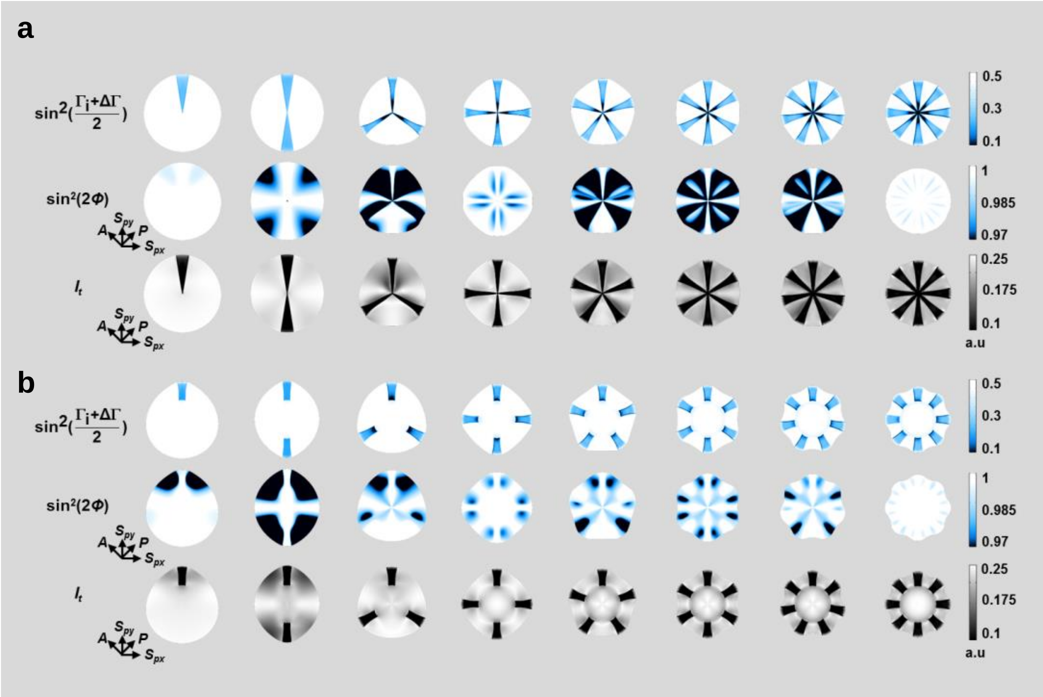

Supplementary Fig. 5 FEM simulation results of $\sin^2((\Gamma_i+\Delta\Gamma)/2)$ and $\sin^2(2\Phi)$ and the optical transmittance (I_t) with crossed polarizers (a) Radial patterns without a central facet and (b) radial patterns with a central facet (D = 2 cm). The gray maps indicate values for $\sin^2((\Gamma_i+\Delta\Gamma)/2)$ and $\sin^2(2\Phi)$ and the optical transmittance (I_t) with crossed polarizers according to the types of the radial patterns and facets implemented for the NIR-induced localized 3D curvature.

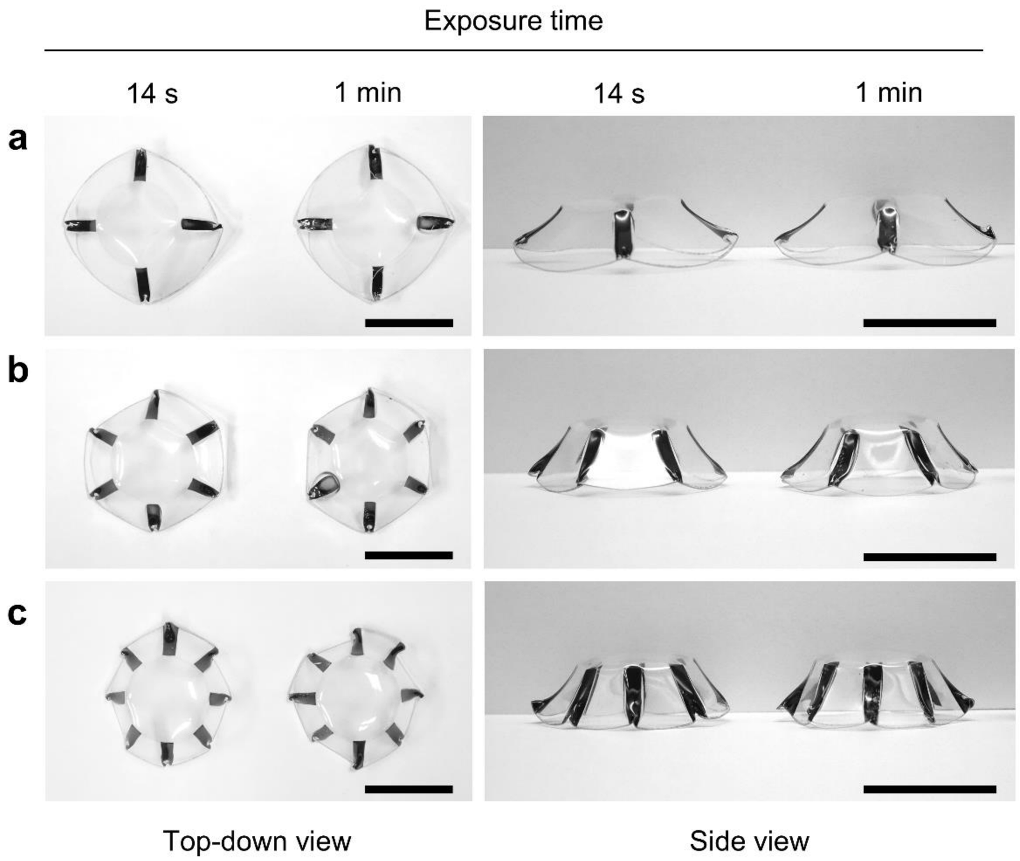

Supplementary Fig. 6 Effect of exposure time on 3D curvature. Resultant 3D shape with comparison of NIR exposure time for 14 s and 1 min. Radial patterns with (a) 4, (b) 6, and (c) 8. Non-patterned central facet region was 2 cm. Left panel showed top-down of curvilinear structure and right panel showed side view after the structure was turned upside down after actuation. Within images, the scale bar depicts 2 cm.

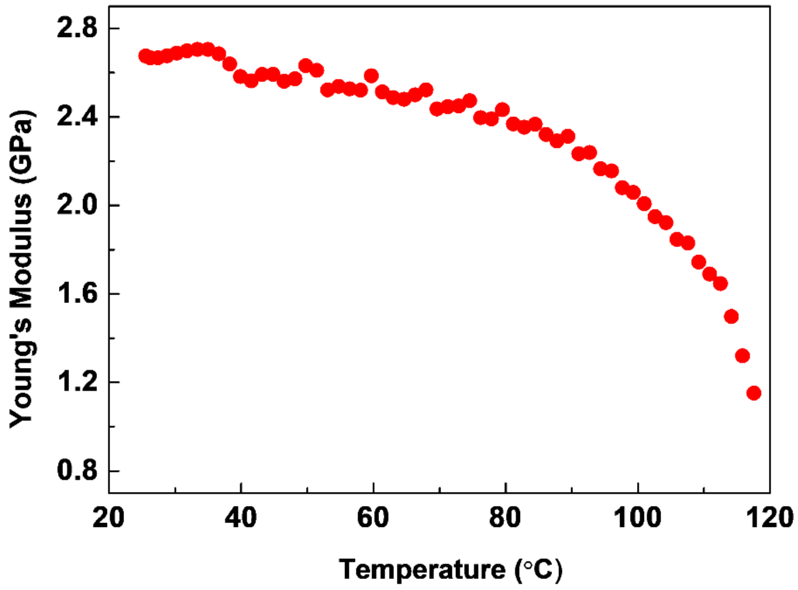

Supplementary Fig. 7 Young's modulus of PS sheet as a function of temperature by dynamic mechanical analyzer (DMA). Young's modulus of 2.7 GPa in glassy region at 26 °C decreased to 1.2 GPa at 118 °C.

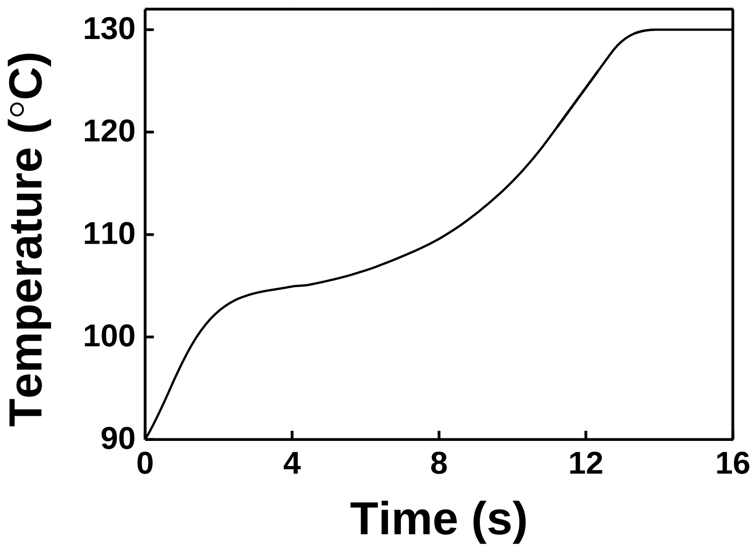

Supplementary Fig. 8 Surface temperature profile of the inked regions according to NIR irradiation time (the NIR intensity was 0.4 W cm^{-2}).

Supplementary Movies

Supplementary Movie 1. Dynamic 3D shape morphing and photo-triggered stress distribution of centro-symmetric 3D curvature (N = 8 and D = 2 cm).

Supplementary Movie 2. Real-time 3D shape morphing of centro-symmetric 3D curvature (N = 4, 6, 8 and D = 2 cm).

Supplementary Movie 3. Dynamic 3D shape morphing and photo-triggered stress distribution of soft-turtle-shell structure.

Supplementary Movie 4. Dynamic 3D shape morphing and photo-triggered stress distribution of sea-shell structure.

Supplementary Movie 5. Real-time 3D shape morphing of Viking helmet architecture via achiral radial pattern.
